# Concomitant novel *ALK*-*SSH2*, *EML4*-*ALK* and *ARID2*-*ALK*, *EML4*-*ALK* double-fusion variants and confer sensitivity to crizotinib in two lung adenocarcinoma patients, respectively

**DOI:** 10.1186/s13000-022-01212-9

**Published:** 2022-02-10

**Authors:** Hong Tao, Zhe Liu, Jing Mu, Fei Gai, Zhan Huang, Liang Shi

**Affiliations:** 1 Department of Oncology, Beijing Chest Hospital, Capital Medical University, Beijing Tuberculosis and Thoracic Tumor Research Institute, Beijing, China; 2 Department of Pathology, Beijing Key Laboratory for Drug Resistant Tuberculosis Research, Beijing Chest Hospital, Capital Medical University, Thoracic Tumor Research Institute, Beijing Tuberculosis, Beijing, China; 3 Department of Medical Business, Amoy Diagnostics Co., Ltd, Xiamen, China

**Keywords:** Lung adenocarcinoma, Anaplastic lymphoma kinase fusion, *ALK*-*SSH2*, *ARID2*-*ALK*, Targeted therapy

## Abstract

**Introduction:**

Anaplastic lymphoma kinase (*ALK*) gene rearrangements, have been identified in approximately 2-7% of patients with lung adenocarcinoma (LUAD). However, co-occurrence of double *ALK* fusions in one patient was rare. Herein, we reported two Chinese female LUAD patients with confirmed double *ALK* fusion variants by next generation sequencing.

**Case presentation:**

Case 1, a 38-year-old female was diagnosed as peripheral LUAD in left upper lobe with synchronous multiple intrapulmonary metastases (pT2N0M1b, stage IVa). And case 2, a 58-year-old female had left lower lobe primary LUAD and synchronous multiple lung metastases (pT4N2M1b, stage IVa). In both patients, tumor cells displayed strong expression of ALK protein. Genetic profiling by next generation sequencing showed both patients concurrently harbored two types of *ALK* rearrangements. Case 1 had an unreported *ALK*-*SSH2*/*EML4*-*ALK* double fusions, and case 2 had an another novel *ARID2*-*ALK*/*EML4*‐*ALK* double fusions. Both of these patients responded to ALK inhibitor crizotinib.

**Conclusions:**

Our study reported two novel *ALK* fusion partners never reported, which expands the knowledge of *ALK* fusion spectrum and provides insight into therapeutic options for patients with double *ALK* fusions.

## Introduction

Anaplastic lymphoma kinase (*ALK*) rearrangement is a well-known driver present in 3–7% of non-small-cell lung cancer (NSCLC) patients [1]. It create an oncogenic ALK tyrosine kinase, which activates many downstream signaling pathways, leading to increased cell proliferation and survival [2]. In addition, *ALK* rearrangement enriched in younger aged adenocarcinoma patients who never smoked or are light smokers. Up to date, various *ALK* fusion partners have been discovered, including *EML4, KIF5B*, *KLC1*, *TFG*, and others [3, 4]. Ongoing efforts also identified *ALK* fusions in other epithelial malignancies, such as *CLIP1*-*ALK*, *KIF5B*-*ALK*, and *KIAA1217*-*ALK*fusions in renal cell carcinoma [5], *BABAM2*-*ALK *fusion in gynecologic clear cell carcinomas [6], *STRN*-*ALK*, *PPP1R21*-*ALK*, and *SENPF*-*ALK *fusions in colorectal carcinoma [7], and *STRN*-*ALK*, and *TPM1*-*ALK *fusions in peritoneal mesothelioma [8]. With the popularity of next-generation sequencing (NGS) technology, more and more rare rearrangement types will be discovered. Application of ALK targeting tyrosine kinase inhibitors such as crizotinib [9], ceritinib [10], alectinib [11], and brigatinib [12], have largely improved prognosis as well as life quality of *ALK* fusion-positive NSCLC patients.

Although novel *ALK* fusion variants have been occasionally reported in NSCLC, to our knowledge, the concomitance of double *ALK* fusion variants in the same lung adenocarcinoma (LUAD) patient was rare, by far, only 6 cases were reported, median age was 44 (range, 29–64 years). The majority of these patients were ever, or current smokers (4/5, 80%) (Table 1) [13–18]. Notably, different fusion partners can impact the response to ALK inhibition in patients with NSCLC [19, 20]. Limited evidence is available on the response to TKI treatment from patients with double *ALK* fusions. Here we reported two novel *ALK* fusion variants concurrently with *EML4*-*ALK* in two LUAD patients respectively (both were female and non-smoker), and both of the patients were sensitive to crizotinib treatment.

**Table 1:** Summary of the characteristics of patients with double *ALK* fusions reported in previous case reports

Ref.	Study	Age	Gender	Smoking	ALK fusion variants	Response to crizotinib
[13]	Xuan Wu, et al. 2020	32	Male	Smoker	*CCNY*-*ALK*, and *ATIC*-*ALK*	Yes
[14]	Xueqian Wu, et al. 2020	64	Female	Non-smoker	*NLRC4*-*ALK*, and *EML4*‐*ALK*	Yes
[15]	Jing Luo, et al. 2019	44	Male	Smoker	*PRKCB*-*ALK*, and *EML4*-*ALK*	Yes
[16]	Bao Dong Qin, et al. 2019	29	Male	Smoker	*EML4*-*ALK*, and *BCL11A*-*ALK*	Yes
[17]	Hao Lin, et al. 2018	56	Male	Not available	*EML6*-*ALK*, and *FBXO11*-*ALK*	Yes
[18]	Jinping Yin, et al. 2018	44	Male	Smoker	*DYSF*-*ALK*, and *ITGAV*-*ALK*	Yes

## Case report

### Case 1

**Fig. 1:**
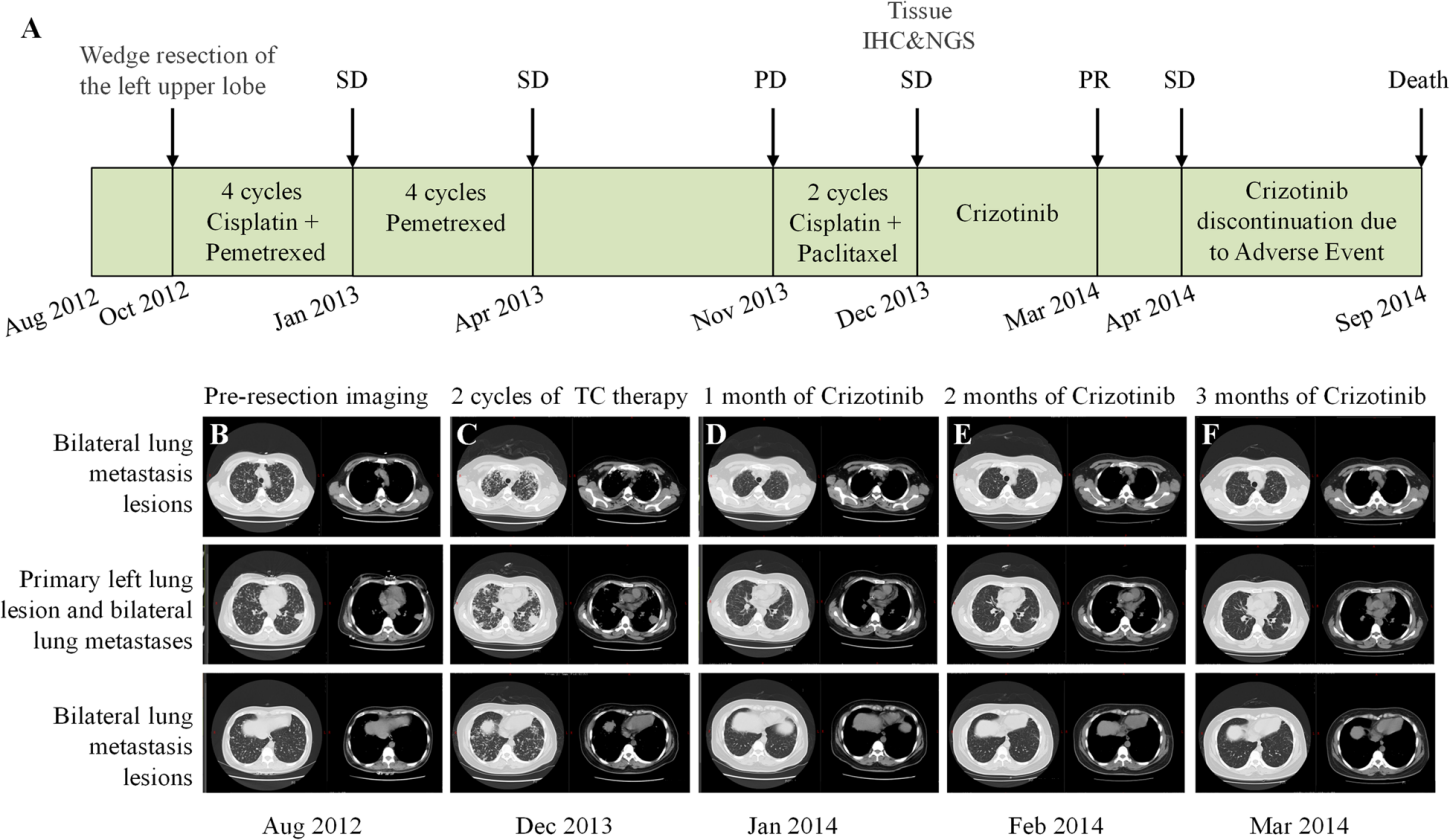
Timeline of the case 1’s disease from diagnosis to death **(A)**. Chest computed tomography (CT) scans at diagnosis **(B)**, and after 2 cycles of Cisplatin + Paclitaxel **(C)**, subsequent chest CT scans performed 1 month **(D)**, 2 months **(E)** and 3 months **(F)** after initiation of crizotinib treatment

**Fig. 2:**
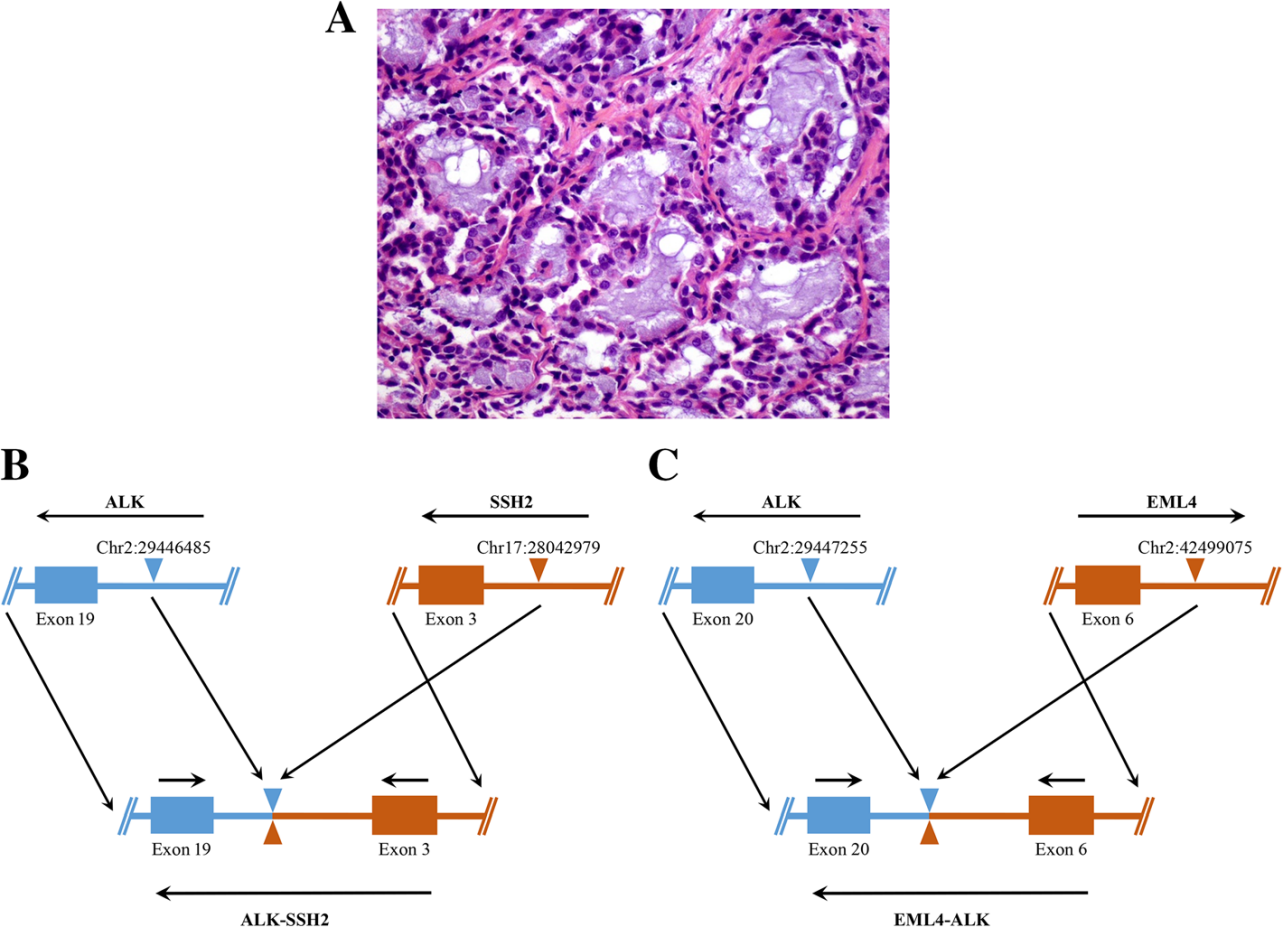
Pathological examination and schematic diagram of *ALK* rearrangement of case 1. **(A)** Hematoxylin and eosin (H&E) staining of the left upper lobe revealed adenocarcinoma (× 400). **(B)** A breakpoint within *ALK* (shown in blue) intron 19 at chromosome 2 was fused within *SSH2* (shown in red) intron 2 at chromosome 17, giving rise to the *ALK*-*SSH2* fusion gene. **(C)** A breakpoint within *ALK* (shown in blue) intron 19 at chromosome 2 was fused within *EML4* (shown in red) intron 6 at chromosome 2, but have opposite orientations, giving rise to the *EML4*-*ALK* fusion gene

The patient was a 38-year-old Chinese female without a history of smoking. The timeline of her diagnosis and treatment was shown in Fig. 1 A and the detailed description was as follows. In 2008, during a routine physical examination in our hospital, chest radiography revealed a left lung shadow without defined diagnosis. In August 2012, a computed tomography (CT) scan revealed a mass in the upper lobe of the left lung with bilateral multiple intrapulmonary metastases (Fig. 1B). This patient underwent thoracoscopic left upper lobectomy on September 12th, 2012. Postoperative diagnosis showed left upper lung adenocarcinoma, pT2N0M1b, stage IVa (Fig. 2 A). Three months after surgery, from January 2013 to October 2013, she received pemetrexed plus cisplatin chemotherapy for 4 cycles, pemetrexed maintenance treatment for 4 cycles, and the diseases remained stable. Disease progressed ​with increased lung lesions in December 2013 (fourteen months after surgery) (Fig. 1 C). Subsequently, this patient received 2 cycles of chemotherapy of paclitaxel plus cisplatin. To seek more potential treatments, in December 2013, immunohistochemistry was performed in postoperative formalin fixed paraffin-embedded (FFPE) tissues, and the tumor cells were positive for ALK D5F3 (Ventana Medical Systems Inc., Oro Valley, AZ, USA). Subsequently, next-generation sequencing (NGS) in a range of 76 cancer-related genes was performed in the FFPE specimen (DNA-based detection, Amoy Diagnostics, Xiamen, China). Revealing coexistence of double *ALK* fusion: *EML4*-*ALK* (E6:A20, MAF = 24.7%) and *ALK*-*SSH2* (A19:S3, MAF = 0.85%) (Fig. 2B), which was further validated by another NGS 10 cancer‐related gene panel (RNA-based detection for fusion genes, Amoy Diagnostics, Xiamen, China), *EML4*-*ALK ALK*-*SSH2* (E6:A20, MAF = 25.6%; A19:S3, MAF = 0.68%, respectively) (Fig. 2 C). This patient was treated with crizotinib (250 mg twice daily) staring from December 31th, 2013. Chest CT scan after one-month treatment indicated a partial response (PR) to crizotinib (Fig. 1D). Dosage of crizotinib was gradually de-escalated to 250 mg once a day in the following 2 months due to increased myocardial enzyme, and the lesions continued to shrink (Fig. 1E). After 2 months of targeted therapy, in March 2014, chest CT showed that the lesion in the left lung was slightly enlarged, and the metastasis in both lungs had no obvious change from the last time (Fig. 1 F). However, crizotinib was discontinued on April 7th, 2014 due to treatment-related adverse events, such as myocardial damage, gastrointestinal reactions, and visual disturbance. This patient died in September 2014 attributing to disease progressing, and having attained an overall survival of 24.9 months.

### Case 2

**Fig. 3:**
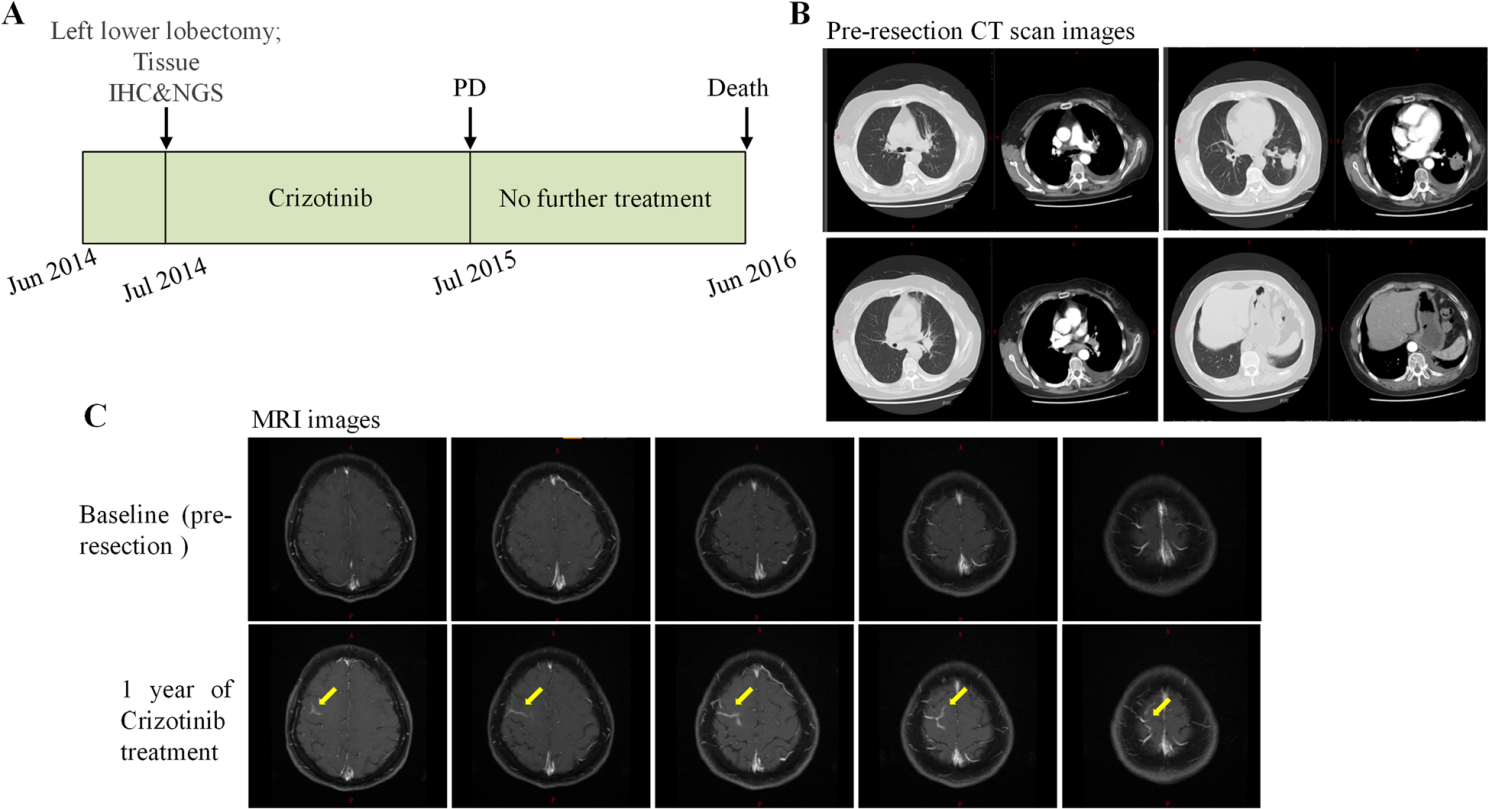
Timeline of the case 2’s disease from diagnosis to death **(A)**. Chest computed tomography (CT) scans at diagnosis **(B)**, and brain magnetic resonance imaging images at baseline (upper panel) and after crizotinib treatment (lower panel) **(C)**. The yellow arrow indicates a new meningeal metastasis lesion

**Fig. 4:**
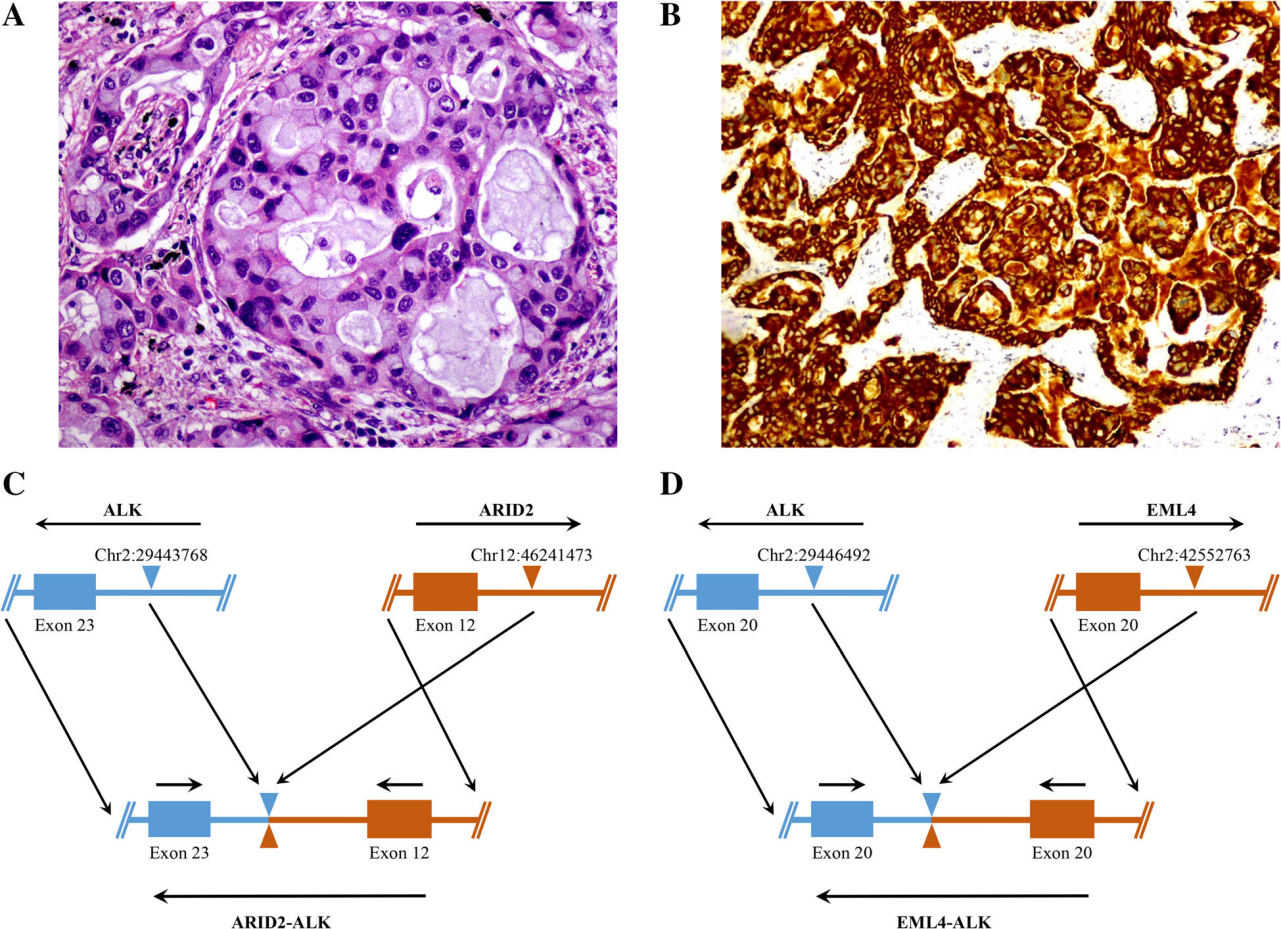
Pathological examination, immunostaining of ALK in postoperative tumor tissue and schematic diagram of *ALK* rearrangement of case 2. **(A)** H&E staining of the left lower lobe revealed adenocarcinoma (×400). **(B)** Immunostaining for ALK D5F3 with strong granular cytoplasmic staining (×200). **(C)** A breakpoint within *ALK* (shown in blue) intron 22 at chromosome 2 was fused within *ARID2* (shown in red) intron 12 at chromosome 12, giving rise to the *ARID2*-*ALK* fusion gene. **(D)** A breakpoint within *ALK* (shown in blue) intron 19 at chromosome 2 was fused within *EML4* (shown in red) intron 20 at chromosome 2, but have opposite orientations, giving rise to the *EML4*-*ALK* fusion gene

This patient was a 58-year-old Chinese female with no history of smoking. The timeline of her diagnosis and treatment was shown in Fig. 3 A and the detailed description was as follows. She was referred to our hospital in June 2014, complaining of breath shortness. A chest CT scan revealed a mass (4.5 × 3.0 × 3.0 cm) in the left lower lobe with bulky swollen mediastinal and multiple nodules in bilateral lung lobes (Fig. 3B). On July 11th, 2014, this patient underwent left lower lobectomy with multifocal resection. Pathological diagnosis indicated a typical lung adenocarcinoma (pT4N2M1b, stage IVa) (Fig. 4 A). Immunohistochemistry of ALK D5F3 (Ventana Medical Systems Inc., Oro Valley, AZ, USA) was positive (Fig. 4B). A NGS analysis to the FFPE specimen using a 76 cancer-related gene panel (DNA-based detection, Amoy Diagnostics, Xiamen, China) revealed a double *ALK* fusion: *EML4*-*ALK ARID2*-*ALK* (E20:A20, 0.99% abundance; and A12:A23, 1.67% abundance) (Fig. 4 C), which was confirmed by another NGS 10 cancer‐related gene panel (RNA-based detection for fusion genes, Amoy Diagnostics, Xiamen, China), the results also showed a double *ALK* fusion: *EML4*-*ALK ARID2*-*ALK* (E20:A20, 0.81% abundance; and A12:A23, 1.09% abundance) (Fig. 4D). The patient was treated with crizotinib 250 mg twice daily in July 31th, 2014 (20 days after surgery), the disease was progressed​ with meningeal metastasis after 12 months’ treatment duration (Fig. 3 C). Unfortunately, she was unable to receive any further treatment due to financial issue. Finally, the patient died in June 28th, 2016, having attained an overall survival of 23.9 months.

## Discussion

*ALK* gene rearrangement is an important driving oncogene in NSCLC. Several different forms of *ALK* fusions have been reported, including *EML4*-*ALK*, the most common *ALK* fusion in NSCLC, which contains the 5’ end of *EML4 *fused to the entire ALK kinase domain and leads to constitutive ligand independent kinase activation [1]. However, the patients who harbor double *ALK* fusion variants are extremely rare. Few investigations have focused on the concomitance of double *ALK* rearrangements because of the low incidence. According to our literature search results, only Six cases have been previously reported, including *CCNY*-*ALK*, and *ATIC*-*ALK *[13], *NLRC4*-*ALK*, and *EML4*‐*ALK *[14], *PRKCB*‐*ALK* and *EML4*‐*ALK *[15], *EML4*‐*ALK* and *BCL11A*‐*ALK*[16], *EML6*-*ALK*, and *FBXO11*-*ALK *[17], as well as *DYSF*‐*ALK* and *ITGAV*‐*ALK *[18]. ALK‐TKIs have been widely used for *ALK*‐positive patients, but the responses are heterogeneous for patient with different *ALK* fusions. The clinical‐pathological characteristics and the response to ALK‐TKIs of such patients with double fusion variants remain unclear, the effectiveness of ALK‐TKI treatment might be affected by the two kinds of *ALK* mutations exist simultaneously in one patient. Here, we identified two novel double *ALK* fusion variants in two LUAD patients respectively, both patients were non-smoking female, one was 38 years old and the other was 58 years old at the time of diagnosis.

Previous reports confirmed that patients with double *ALK *fusion may respond to crizotinib [14–18]. In this report, the patients with double *ALK* fusions were also sensitive to crizotinib, and two novel *ALK* fusions: ALK-*SSH2* and *ARID2*-*ALK* were detected. Case 1 was diagnosed at advanced stage and received multiline chemotherapy, without condition improvement; instead, bone metastases developed. Except *EML4*-*ALK*, *ALK*-*SSH2 *was uncovered. Slingshot 2 (SSH2) belongs to a gene family of three members (SSH1, SSH2, and SSH3), it has been shown to control some essential cellular processes, including invasion, migration, and motility [21]. In *ALK*-*SSH2* fusion, the fusion point falls into intron 2 of *SSH2* and the promoter region was retained. As for *ALK*, the entire intracellular kinase domain was retained. And it is well known that the fusion leading to constitutive kinase activation can be a powerful driving force for oncogenesis. Thus, it is speculated that the concomitance of *ALK*-*SSH2* fusion maybe one of the reasons for sensitivity to crizotinib. Case 2 with another novel *ALK* fusion variant, *ARID2*-*ALK*, was presented a SD to crizotinib for about 1 year, and progressed with meningeal metastasis. *ARID2* encode nuclear proteins containing a DNA-binding domain called AT‐rich interaction domain (ARID domain), and is implicated in chromatin remodeling. *ARID2 *was found mutated in hepatocellular carcinoma [22], melanoma [23] and lung carcinoma [24]. Given that the mutation abundance of *EML4*-*ALK* was very low (less than 1%), so it is speculated that the coexistence of *ARID2*-*ALK* fusion maybe one of the reasons for response to crizotinib in this patient.

The most common adverse events in clinical trials with crizotinib were visual disorders, nausea–vomiting, diarrhea, edema, and elevated transaminases [25]. Due to the increasing clinical experience with crizotinib, other toxicities are emerging, such as QT interval prolongation, bradycardia, hypogonadism, renal impairment, renal cysts and hypersensitivity [26–29]. The treatment-related adverse events that occurred in case 1 had been reported in aforementioned clinical trials. After reducing the drug dose, grade 1-2 adverse events including diarrhea and visual disturbance were all alleviated. Unfortunately, crizotinib treatment was eventually stopped due to un-tolerable adverse events in case 1, mainly because of the myocardial damage.

Case 2 remained stable during crizotinib treatment, but brain metastasis occurred after 1 year of treatment. As a drive gene mutation, *ALK* gene rearrangement accounts approximately 7% of all cases of NSCLC. And these patients achieve prolonged PFS when treated with crizotinib, a first-generation ALK-targeted tyrosine kinase inhibitor. However, most patients experience tumor recurrence within 1 year after crizotinib therapy. Moreover, brain metastasis, which remains a substantial cause of morbidity and mortality, is the most common type of recurrence [30, 31].

In this study, both patients responded to crizotinib treatment. The first patient gave up the treatment due to intolerable treatment-related adverse events, while the second patient occurred brain metastasis after one year of treatment, and also gave up the follow-up due to economic reasons. Since there were no specific research results to support, we can only speculate that coexistence of double *ALK* fusion may be related to the occurrence of serious adverse events or drug resistance.

There are some limitations in our present study. First, the case 2 did not receive subsequent treatment such as chemotherapy and radiotherapy, which might have an impact on her overall survival. Second, the biological function of *ALK*-*SSH2* and *ARID2*-*ALK* should be further investigated using cell lines and animal models after molecular manipulation of *ALK*-*SSH2* and *ARID2*-*ALK*.

In conclusion, this study is described two novel *EML4*-*ALK ALK*-*SSH2*, and *EML4*-*ALK ARID2*-*ALK* double *ALK* fusion variants LUAD patients. And the curative effect of crizotinib in the treatment of these patients provided a certain reference for the patients with such gene alterations. In addition, the NGS assay provides a reliable diagnostic tool for the detection of novel fusion partner genes for *ALK*-rearranged in patients with lung adenocarcinoma.

## Data Availability

All data generated or used during the study are available from the corresponding author by request.
